# Supplementary material for: Chest Computed Tomography Characteristics of Critically Ill COVID-19 Patients with Auto-antibodies Against Type I Interferons
Source: J Clin Immunol. 2023 Dec 22;44(1):15. doi: 10.1007/s10875-023-01606-4 (PMC10739505; doi:10.1007/s10875-023-01606-4)
Supplement: Supplementary file 1 — (DOCX 24 kb) [file 10875_2023_1606_MOESM1_ESM.docx]

**Chest computed tomography characteristics of critically ill COVID-19 patients with auto-antibodies against type I interferons**

**Supplementary document**

**Table of content**

Supplementary Table 1……………………………………………………………………………………………………………………..2

Supplementary Table 2……………………………………………………………………………………………………………………..3

**Table S1. Clinical characteristics and outcomes of patients with (n=48) or without (n=342) type I anti-interferon auto-antibodies**

|  | **Anti-IFN auto-Abs**  **n=48** | **No anti-IFN auto-Abs n=342** |
| --- | --- | --- |
| Age (mean, SD) | 59.0 (13.2) | 60.0 (12.2) |
| Male | 39 (81.2) | 240 (70.2) |
| BMI | 29.3 (5.6) | 30.6 (5.9) |
| Clinical Frailty scale | 29.0 (5.6) | 30.0 (5.8) |
| Comorbidities |  |  |
| Diabetes | 15 (31.2) | 102 (29.8) |
| Hypertension | 21 (43.8) | 173 (50.6) |
| Chronic cardiac failure | 6 (12.5) | 44 (12.9) |
| Chronic kidney disease | 4 (8.3) | 38 (11.1) |
| Cirrhosis | 0 (0.0) | 6 (1.8) |
| COPD / Chronic respiratory failure | 2 (4.2) | 21 (6.1) |
| Asthma | 3 (6.2) | 24 (7.0) |
| Immunosuppression |  |  |
| HIV infection | 0 (0.0) | 7 (2.0) |
| Solid organ transplant | 1 (2.1) | 22 (6.4) |
| Hemopathy | 0 (0.0) | 4 (1.2) |
| Chronic corticosteroid treatment | 1 (2.1) | 20 (5.8) |
| Auto-immune disease | 2 (4.2) | 14 (4.1) |
| History of intrathoracic disease |  |  |
| Pneumonia | 5 (10.4) | 15 (4.4) |
| Tuberculosis | 0 (0.0) | 7 (2.1) |
| Pleural effusion | 1 (2.1) | 2 (0.6) |
| Neoplasia | 1 (2.1) | 7 (2.1) |
| Cervical or thoracic radiotherapy | 1 (2.1) | 8 (2.3) |
| Pneumotoxic drug exposure | 3 (6.2) | 52 (15.2) |
| Admission data |  |  |
| Days between symptoms and ICU admission | 10.0 (6.0 ; 14.0) | 9.0 (6.0 ; 12.0) |
| Days between ICU admission and chest CT† | 0.0 (-1.8; 0.8) | -1.0 (-2.0; 0.0) |
| WHO severity scale | 7.0 (6.0 ; 8.0) | 6.0 (6.0 ; 8.0) |
| Steroid treatment for COVID-19 | 17 (35.4) | 181 (52.9) |
| Tocilizumab | 2 (4.2) | 34 (9.9) |
| SOFA | 4.0 (2.0 ; 6.0) | 4.0 (2.0 ; 5.0) |
| Outcome |  |  |
| Deceased at day 90 | 14 (29.2) | 102 (29.8) |

Results are for the entire cohort, whether they had a chest CT scan acquired or not. Results are N(%), means (±standard deviation) or medians (interquartile range); SD: standard deviation; BMI: body mass index in kg/m²; COPD: chronic obstructive pulmonary disease; HIV: human immunodeficiency virus; ICU: intensive care unit; WHO: World Health Organization; COVID-19: coronavirus disease 19; ECMO: extracorporeal membrane oxygenation; SOFA: sequential organ failure assessment; IFN: interferon; Abs: antibodies.

†: n= 231 patients with chest CT available

**Table S2. Characteristics of patients with or without a chest CT study available.**

|  | **CT study**  **n=244** | **No CT study**  **n=166** | **p-value** |
| --- | --- | --- | --- |
| Age (mean, SD) | 59.5 (12.7) | 60.0 (12.3) | 0.844 |
| Male | 182 (74.6) | 111 (66.9) | 0.096 |
| BMI | 29.3 (5.6) | 30.6 (5.9) | **0.026** |
| Frailty scale | 3.0 (2.0-3.0) | 3.0 (2.0-4.0) | 0.671 |
| Comorbidities |  |  |  |
| Diabetes | 69 (28.3) | 54 (32.5) | 0.381 |
| Hypertension | 109 (44.7) | 93 (56.0) | **0.027** |
| Chronic cardiac failure | 31 (12.7) | 20 (12.0) | 0.880 |
| Chronic kidney disease | 21 (8.6) | 22 (13.3) | 0.142 |
| Cirrhosis | 3 (1.2) | 3 (1.8) | 0.690 |
| COPD / Chronic respiratory failure | 14 (5.7) | 10 (6.0) | 1.000 |
| Asthma | 10 (4.1) | 18 (10.9) | **0.009** |
| Immunosuppression |  |  |  |
| HIV infection | 3 (1.2) | 5 (3.0) | 0.278 |
| Solid organ transplant | 12 (4.9) | 11 (6.6) | 0.515 |
| Hemopathy | 3 (1.2) | 1 (0.6) | 0.650 |
| Chronic corticosteroid treatment | 14 (5.7) | 7 (4.2) | 0.649 |
| Auto-immune disease | 9 (3.7) | 7 (4.2) | 0.799 |
| History of intrathoracic disease |  |  |  |
| Pneumonia | 9 (3.7) | 12 (7.2) | 0.117 |
| Tuberculosis | 2 (0.8) | 5 (3.0) | 0.124 |
| Pleural effusion | 4 (1.6) | 1 (0.6) | 0.652 |
| Neoplasia | 2 (0.8) | 7 (4.2) | **0.034** |
| Cervical or thoracic radiotherapy | 4 (1.6) | 6 (3.6) | 0.212 |
| Pneumotoxic drug exposure | 28 (11.5) | 28 (17.0) | 0.142 |
| Admission data |  |  |  |
| Days between symptoms and ICU admission | 9.0 (6.0-12.0) | 10.0 (6.0-14.0) | 0.118 |
| WHO severity scale | 6.0 (6.0-8.0) | 8.0 (6.0-8.0) | **<0.001** |
| Steroid treatment for COVID-19 | 128 (52.5) | 78 (47.0) | 0.314 |
| Tocilizumab | 22 (9.0) | 15 (9.0) | 1.000 |
| ECMO | 2 (0.8) | 19 (11.4) | **<0.001** |
| SOFA | 3.0 (2.0-4.0) | 4.0 (2.0-7.0) | **<0.001** |
| Anti-IFN auto-Abs positivity | 30 (13.0) | 18 (11.3) | 0.642 |
| Outcomes |  |  |  |
| Day 28 mortality | 57 (23.4) | 49 (29.5) | 0.170 |
| Day 90 mortality | 72 (29.5) | 51 (30.7) | 0.827 |

Results are N(%), means (±standard deviation) or medians (interquartile range); CT: computed tomography; SD: standard deviation; BMI: body mass index in kg/m²; COPD: chronic obstructive pulmonary disease; HIV: human immunodeficiency virus; ICU: intensive care unit; WHO: World Health Organization; COVID-19: coronavirus disease 19; ECMO: extracorporeal membrane oxygenation; SOFA: sequential organ failure assessment; IFN: interferon; Abs: antibodies; Two-tailed p-values come from unadjusted comparisons using Chi square or Fisher’s exact tests for categorical variables, and t-tests or Mann-Whitney tests for continuous variables, as appropriate; **bolded** variables are significant at the p<0.05 level.
